# Supplementary material for: Genetic characteristics of P. falciparum parasites collected from 2012 to 2016 and anti-malaria resistance along the China-Myanmar border
Source: PLoS One. 2023 Nov 10;18(11):e0293590. doi: 10.1371/journal.pone.0293590 (PMC10637670; doi:10.1371/journal.pone.0293590)
Supplement: S1 File — Photos of relevant content in reference 39. (PDF) [file pone.0293590.s001.pdf]

和初步数据分析。GenAlEx 软件可以帮助我们实现这个目的。这个软件可以从 http://biology-assets.anu.edu.au/GenAlEx/Download.html 这一网址下载。

GenAlEx 软件是在 Microsoft Excel 下使用，要调用宏，因此我们首先要把 Excel 的宏“安全级”设为“中”（图 3-39，图 3-40）。然后点击“GenAlEx 6.501.xla”加载 GenAlEx 这个软件（图 3-41），Excel 提示“启用宏”，点击启用（图 3-42），软件就加载入 Excel 了。作者提供了一个“mg.xls”文件作演示用，其数据格式说明见图 3-43。这个演示文件包括两个种群，如果读者的数据是多个种群，如 10 个种群，那么就把数据第一行的 2 改成 10，然后依次把每个种群的个体数目写在随后的数据格中。

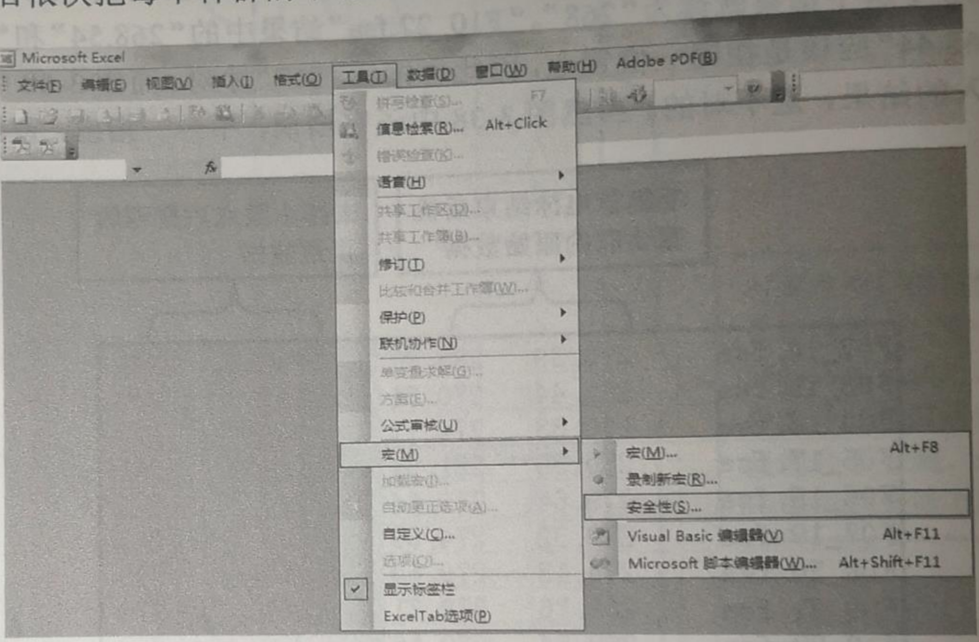

图 3-39　调整 Excel 的宏安全性之一

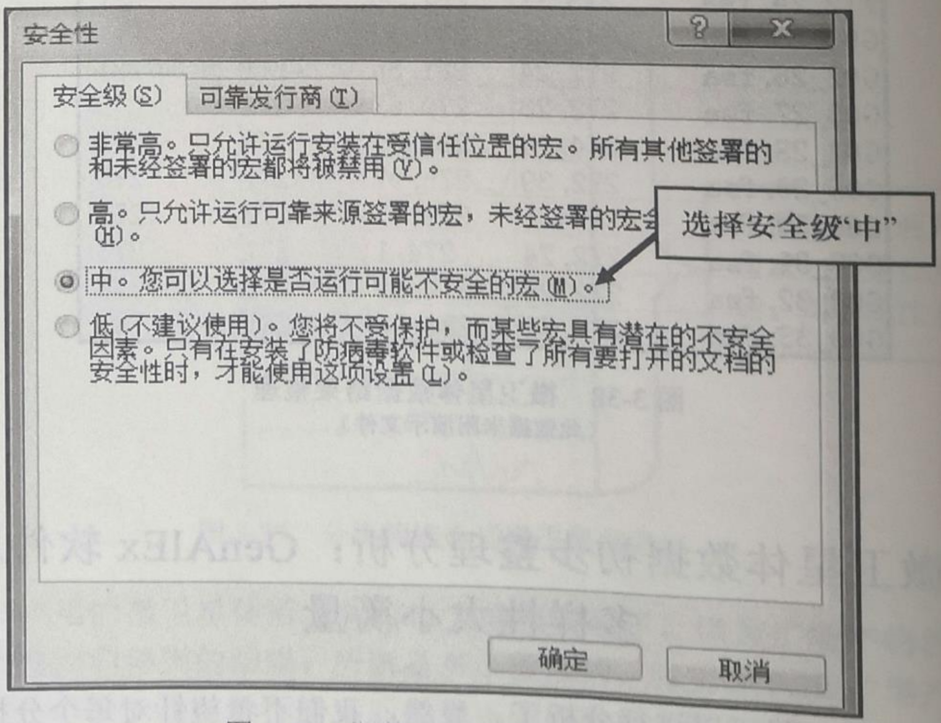

图 3-40　调整 Excel 的宏安全性之二

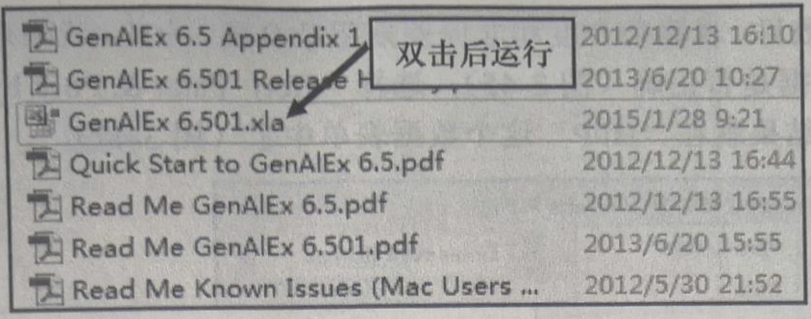

图 3-41　运行 GenAlEx 软件

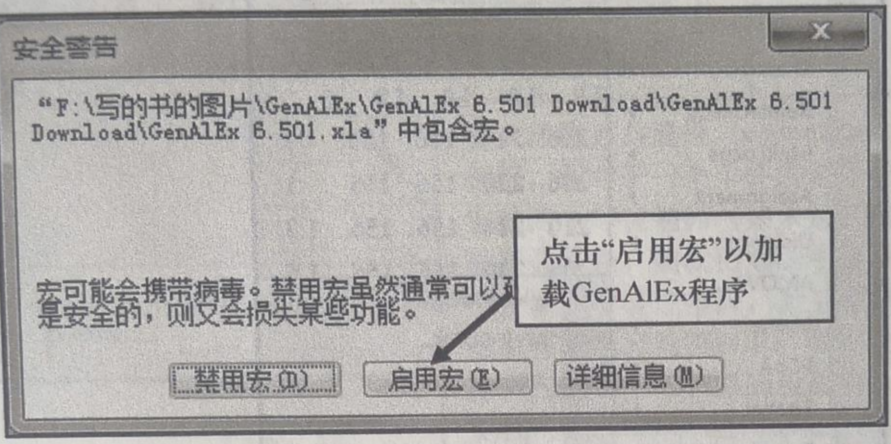

图 3-42　启用宏后在 Excel 中加载 GenAlEx 程序软件

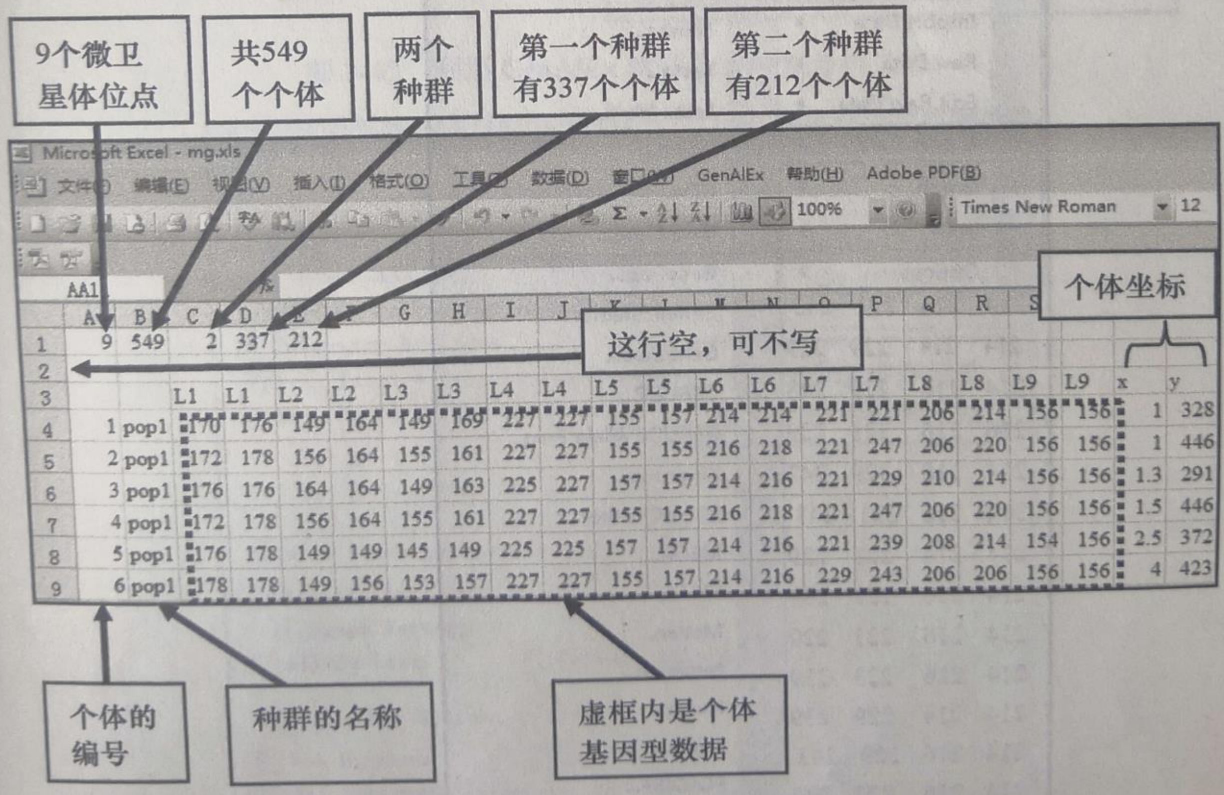

图 3-43　GenAlEx 软件数据格式

GenAlEx 软件提供了非常丰富的内容，可以帮助我们对数据进行初步的分析和多个软件间的格式转换（图 3-44）。作者将用它的 "Export Data" 功能做 AIS 和 Genepop 软件的数据格式转换。在这之前我们可以先看下数据的大致统计结果。我们主要看三个统

计参数：等位基因数目、观测杂合度和非偏差期望杂合度。为此，我们选择“Frequency…”（图 3-44），检查数据是否正确（图 3-45），选择“Het，Fstat & Poly by Pop”（图 3-46）后，点击“OK”，结果就在“HFP”这个数据表单中了（图 3-47）。

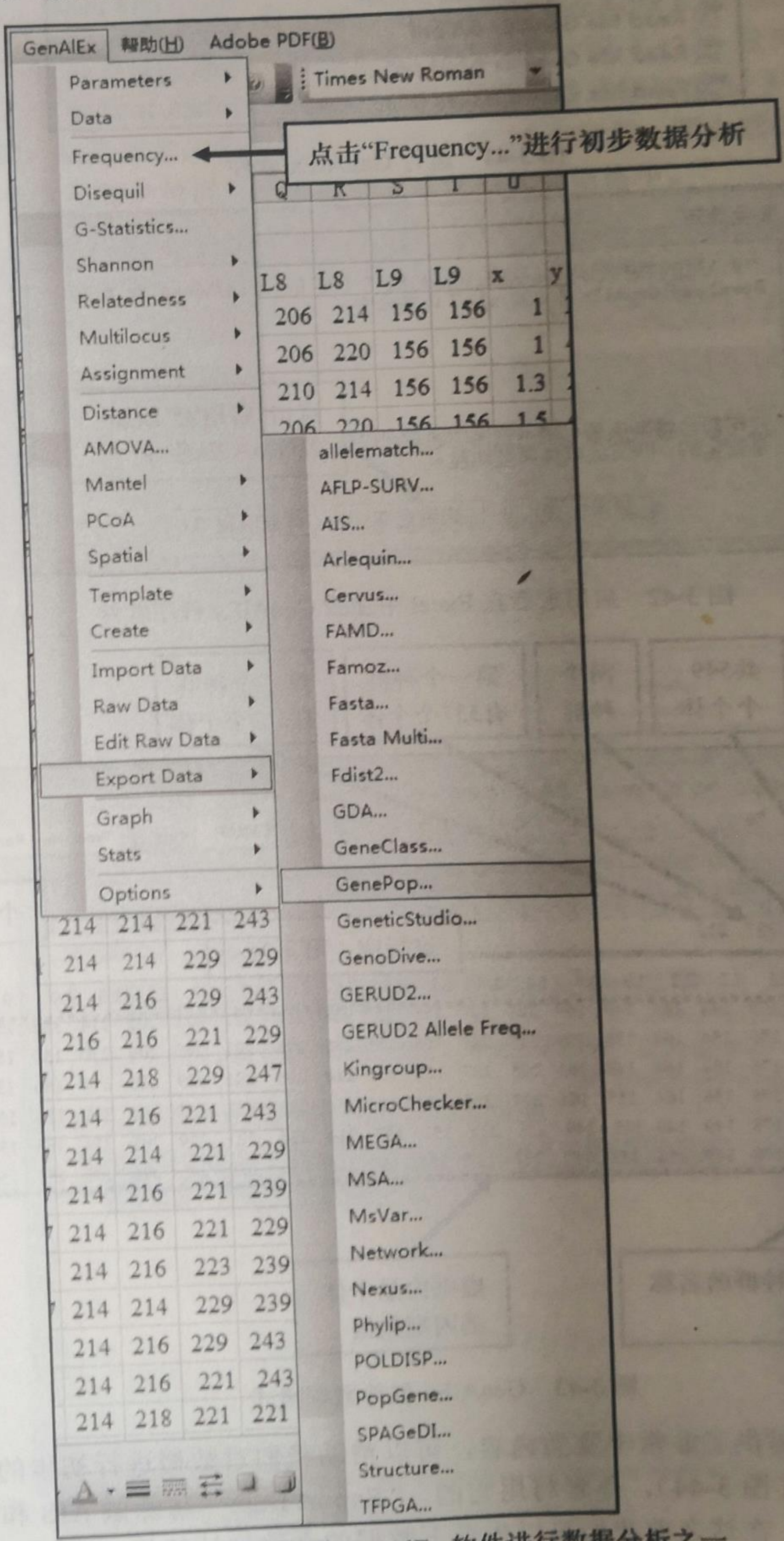

图 3-44 利用 GenAlEx 软件进行数据分析之一

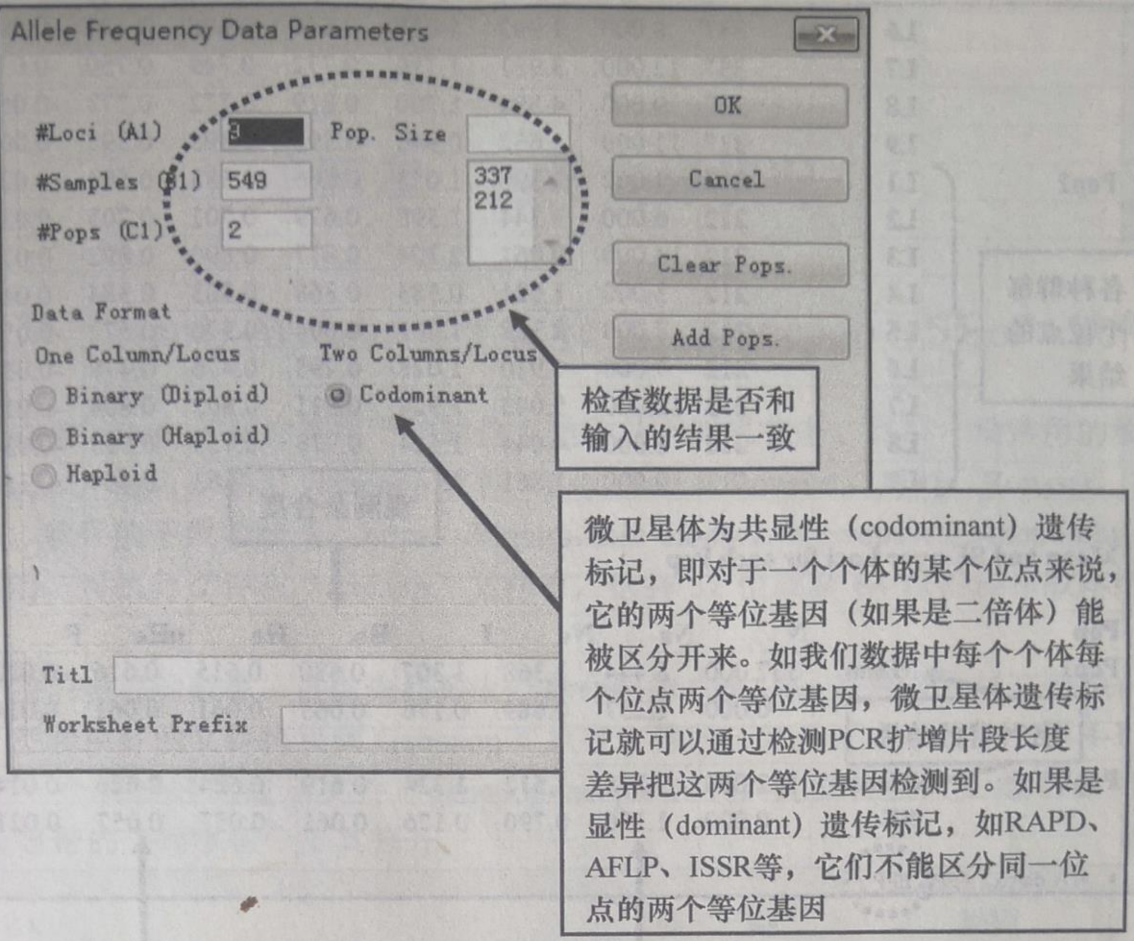

图 3-45　利用 GenAlEx 软件进行数据分析之二

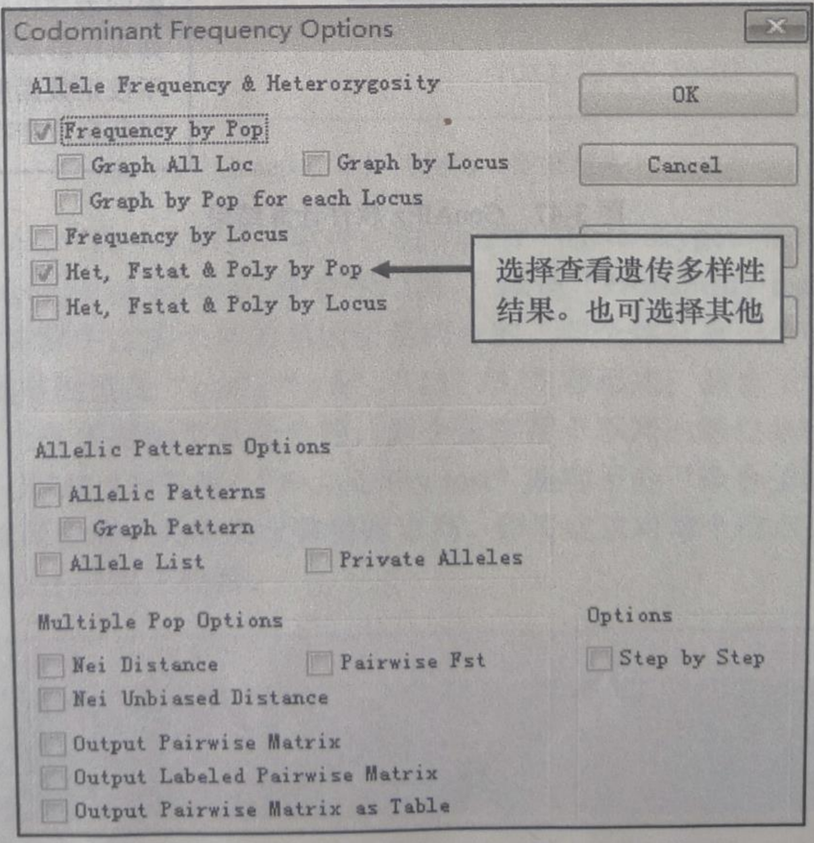

图 3-46　利用 GenAlEx 软件进行数据分析之三

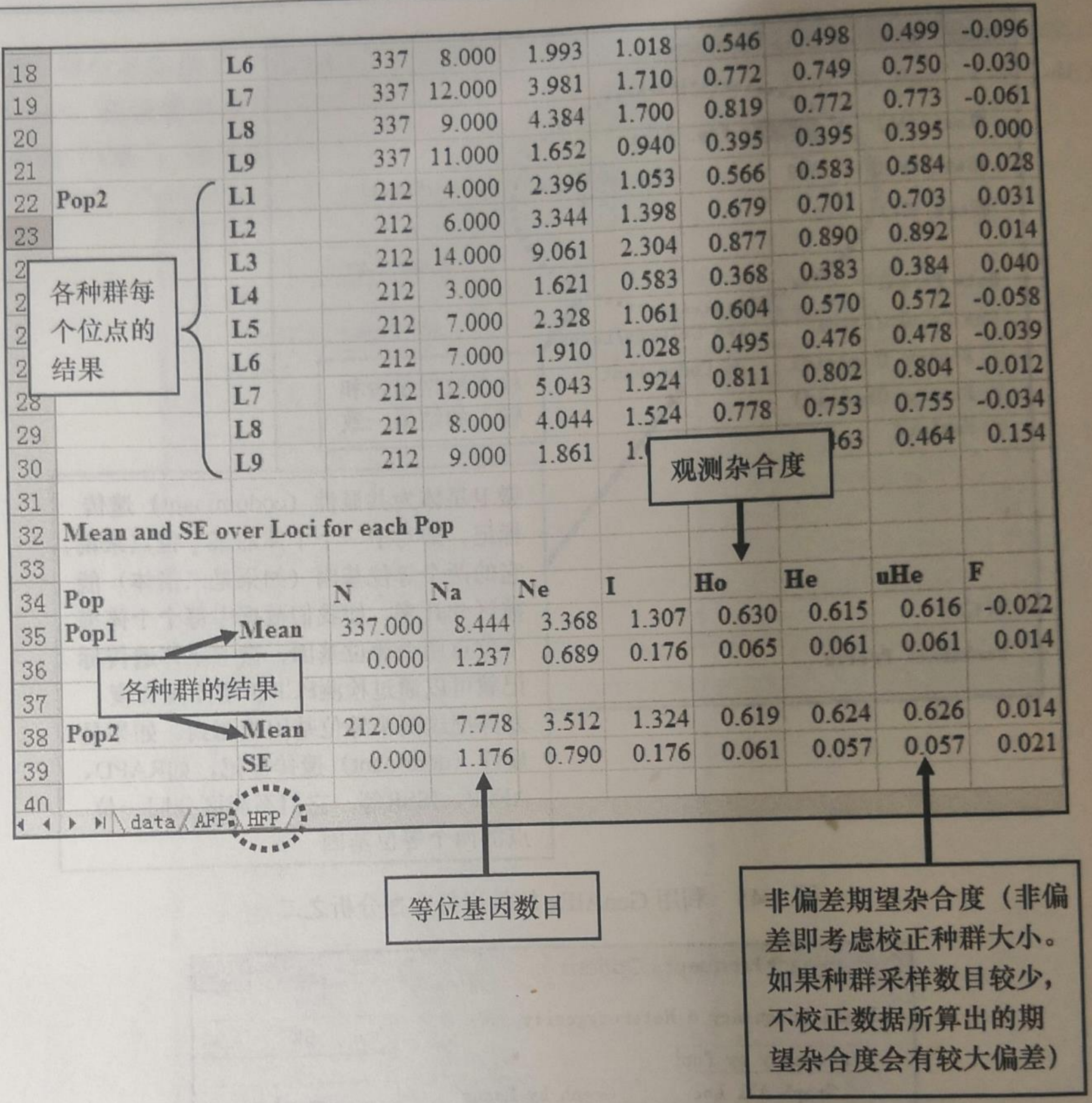

			N	Na	Ne	I	Ho	He	uHe	F
18		L6	337	8.000	1.993	1.018	0.546	0.498	0.499	-0.096
19		L7	337	12.000	3.981	1.710	0.772	0.749	0.750	-0.030
20		L8	337	9.000	4.384	1.700	0.819	0.772	0.773	-0.061
21		L9	337	11.000	1.652	0.940	0.395	0.395	0.395	0.000
22	Pop2	L1	212	4.000	2.396	1.053	0.566	0.583	0.584	0.028
23		L2	212	6.000	3.344	1.398	0.679	0.701	0.703	0.031
2[illegible]		L3	212	14.000	9.061	2.304	0.877	0.890	0.892	0.014
2[illegible]		L4	212	3.000	1.621	0.583	0.368	0.383	0.384	0.040
2[illegible]		L5	212	7.000	2.328	1.061	0.604	0.570	0.572	-0.058
2[illegible]		L6	212	7.000	1.910	1.028	0.495	0.476	0.478	-0.039
28		L7	212	12.000	5.043	1.924	0.811	0.802	0.804	-0.012
29		L8	212	8.000	4.044	1.524	0.778	0.753	0.755	-0.034
30		L9	212	9.000	1.861	1.[illegible]	[illegible]	[illegible]63	0.464	0.154
31										
32	Mean and SE over Loci for each Pop									
33										
34	Pop		N	Na	Ne	I	Ho	He	uHe	F
35	Pop1	Mean	337.000	8.444	3.368	1.307	0.630	0.615	0.616	-0.022
36		[illegible]	0.000	1.237	0.689	0.176	0.065	0.061	0.061	0.014
37										
38	Pop2	Mean	212.000	7.778	3.512	1.324	0.619	0.624	0.626	0.014
39		SE	0.000	1.176	0.790	0.176	0.061	0.057	0.057	0.021
40										

图 3-47　GenAlEx 软件计算结果

第五章 遗传分化

第一节 F_{ST}分析：Genetix 软件

遗传分化是衡量种群间遗传差异的重要参数。有两种最基本的度量值，一种是 F_{ST} 值，另一种是 Φ_{ST} 值。不论 F_{ST} 值还是 Φ_{ST} 值，对于多种群（两个以上种群）来说，它们都可分为总值和两两之间的值。总值是把所有种群总体计算得出的一个总体衡量值，而两两之间的遗传分化值就是每一对种群间计算的值。

首先介绍如何进行 F_{ST} 值的计算。在此介绍使用 Genetix 软件（Belkhir et al., 1996-2004），它可以从 http：//kimura.univ-montp2.fr/genetix/constr.htm#download 下载（这个软件界面是法文的）（图 5-1）。

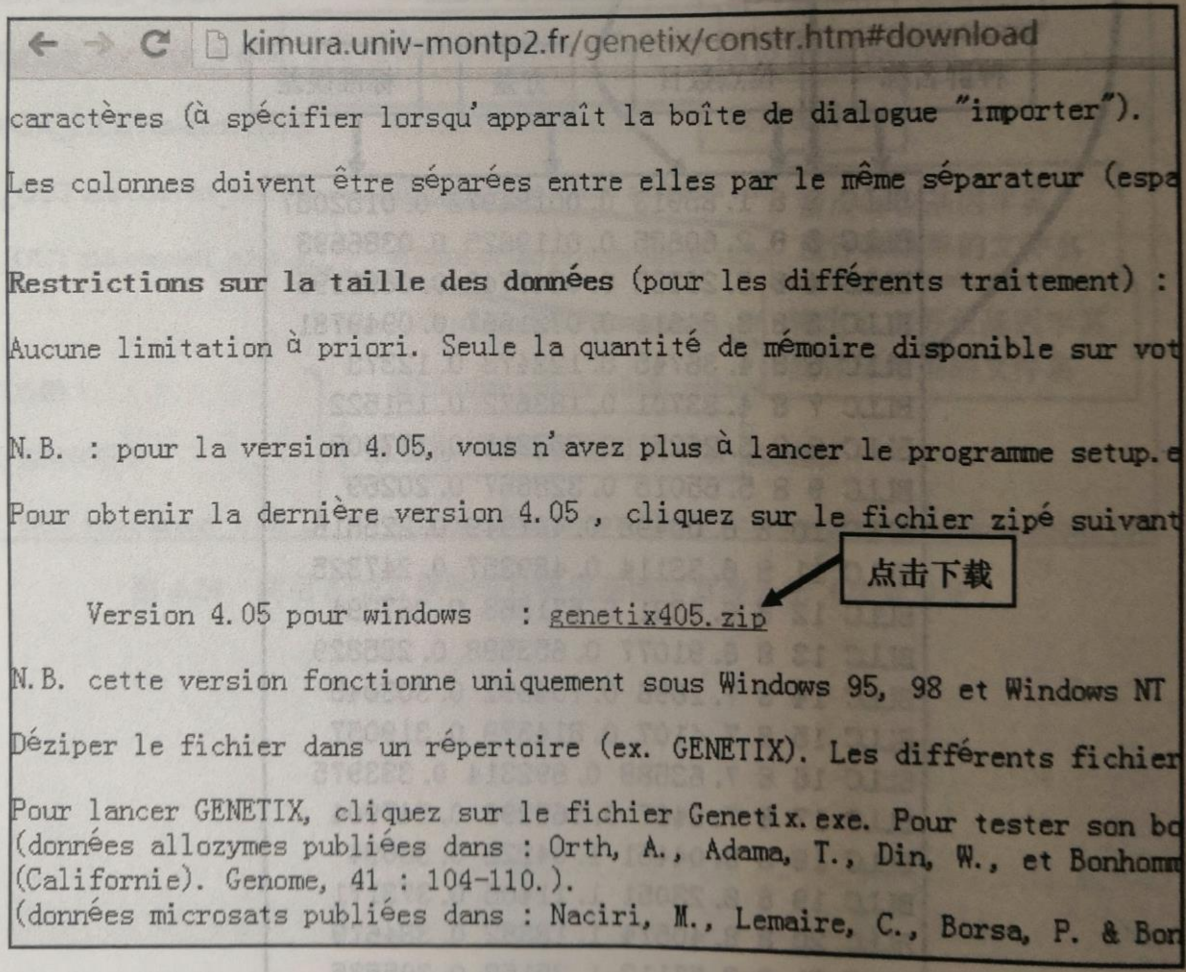

图 5-1 下载 Genetix 软件

下载后解压缩，双击“Genetix.exe”就可使用。Genetix 软件也有自己的数据输入格式，但也可以使用其他软件的格式。可以先用 GenAlEx 软件中的“Export Data”功能把我们的数据转换为“GenePop”格式，然后使用 Genetix 软件中的“Importer”功能把数

两两结果计算后，如对于种群 1 和种群 3，遗传分化 F_{ST} 值为 0.029 75，1000 次模拟结果表明这个值非常显著（P<0.01），因为没有一次模拟结果大于它（0.00%）（图 5-10）。如果两两种群的“% val >”这个结果是 0.02，表示 1000 次模拟结果中只有 0.02%的结果是大于 0.029 75 的，那么 P 值是 0.02，小于 0.05，也是显著的。

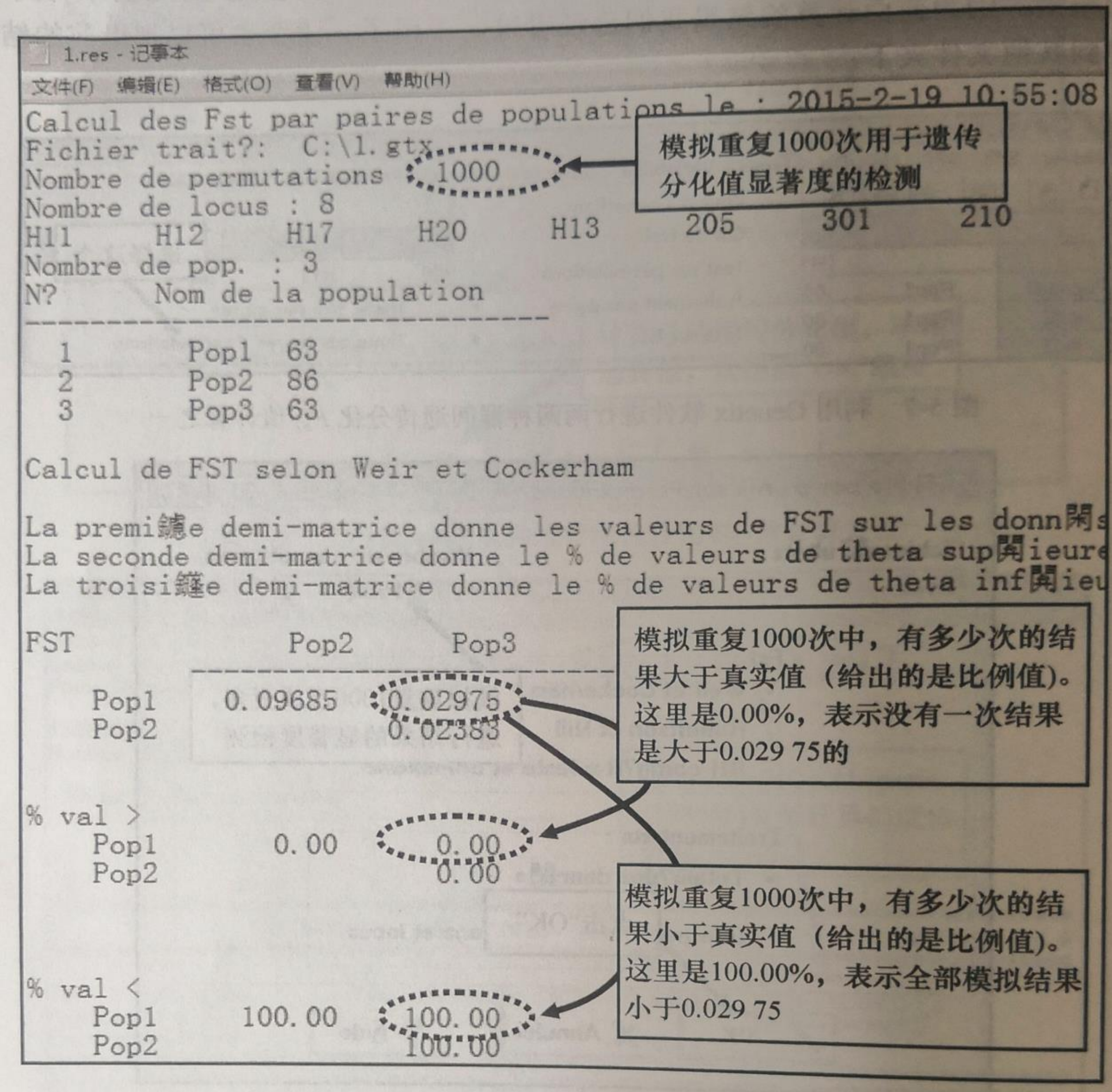

```
1.res - 记事本
文件(F) 编辑(E) 格式(O) 查看(V) 帮助(H)
Calcul des Fst par paires de populations le : 2015-2-19 10:55:08
Fichier trait?: C:\1.gtx
Nombre de permutations 1000
Nombre de locus : 8
H11    H12    H17    H20    H13    205    301    210
Nombre de pop. : 3
N?     Nom de la population
--------------------------------
  1      Pop1  63
  2      Pop2  86
  3      Pop3  63

Calcul de FST selon Weir et Cockerham

La premi鑢e demi-matrice donne les valeurs de FST sur les donn閑s
La seconde demi-matrice donne le % de valeurs de theta sup閑ieure
La troisi鑫e demi-matrice donne le % de valeurs de theta inf閑ieu

FST          Pop2        Pop3
          ----------------------------
   Pop1   0.09685     0.02975
   Pop2               0.02383

% val >
   Pop1      0.00        0.00
   Pop2                  0.00

% val <
   Pop1    100.00      100.00
   Pop2                100.00
```


图 5-10 利用 Genetix 软件计算的两两种群间遗传分化 F_{ST} 值结果

第二节 AMOVA 分析：GenAlEx 软件

衡量种群遗传分化的另外一种方法是分子方差分析（analysis of molecular variance，AMOVA）方法（Excoffier et al.，1992）。不同于 F_{ST} 使用等位基因频率的方法计算种群间遗传分化，AMOVA 方法使用遗传距离（genetic distance）的方法来进行种群间遗传分化的衡量，扩展了遗传分化使用的范围（如个体序列间的差异不仅能用基因频率的方法衡量，还可以用两者之间碱基差异多少进行衡量），使得利用各种分子标记（如 RFLP、RAPD、AFLP、DNA 序列等）都可以进行遗传分化分析。为区别于 F_{ST} 值，AMOVA 用 Φ_{ST} 值来代表。

我们可以使用前面介绍的 GenAlEx 软件进行 AMOVA 分析，操作方便。在此用“1.xls”

据读入（图 5-2，图 5-3）。在此用作者转换好的一个数据“1.gtx”进行演示。这个文件包含了三个种群。首先，我们计算总的遗传分化值。打开文件，然后按图 5-4 和图 5-5 操作。计算完成后软件提示“TRAITEMENT FINI!”说明运算结束，点击“确定”就可以，

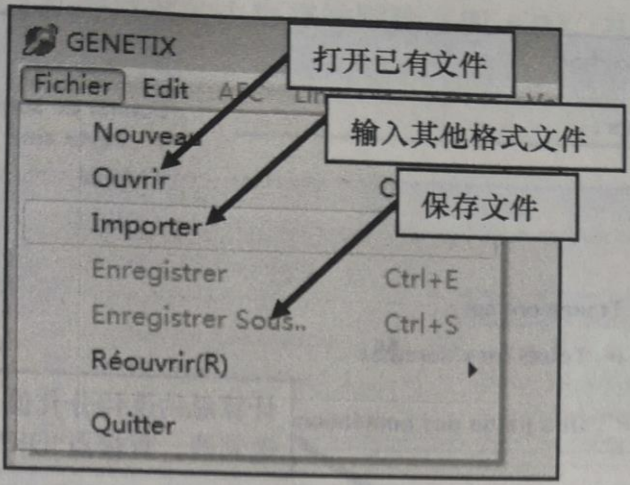

图 5-2 Genetix 软件数据输入、保存

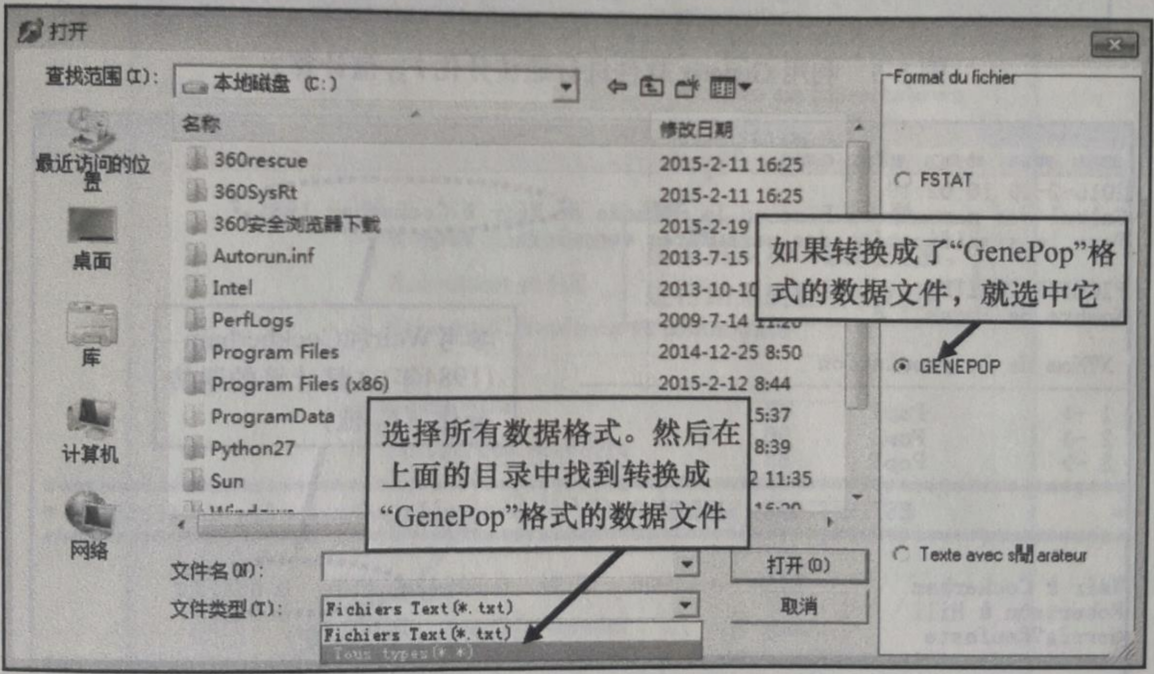

图 5-3 利用 Genetix 软件输入不同格式的文件

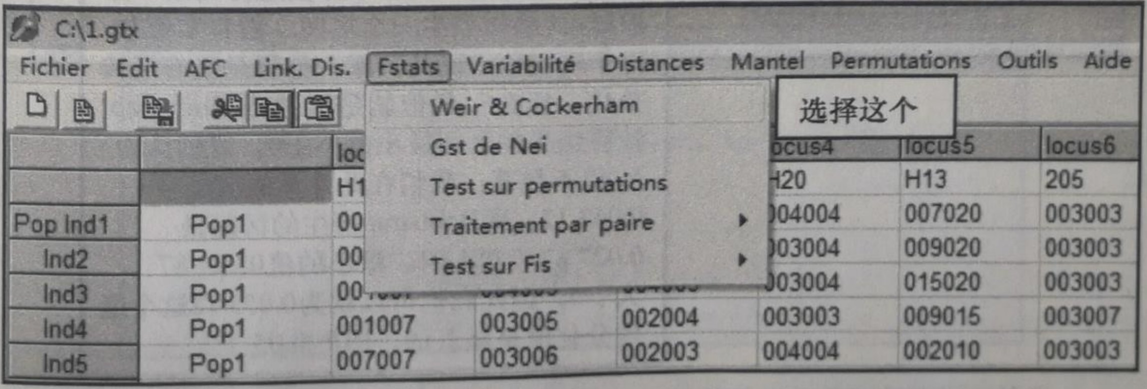

图 5-4 利用 Genetix 软件进行遗传分化 F_{ST} 值计算之一

然后软件自动打开运算的结果（如没有打开，结果文件“1.res”可用“记事本”工具打开），结果文件会自动保存在和数据文件同一文件夹下。例如，作者的数据文件是放在C盘的根目录下（即“C：\”），运算的结果也被放在了这个目录下。结果分析请见图5-6。

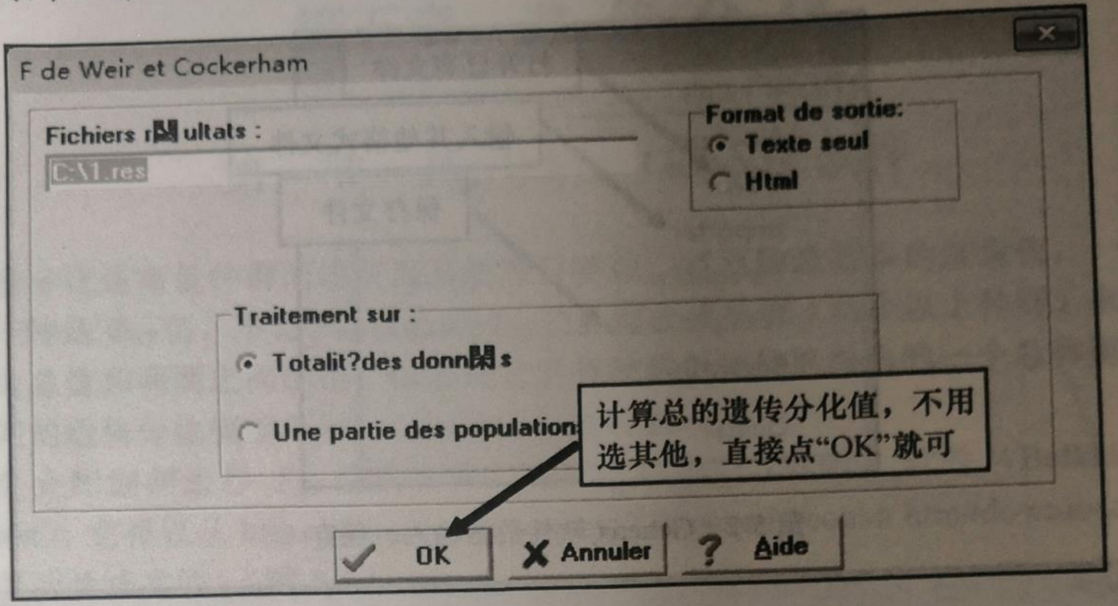

图5-5　利用Genetix软件进行遗传分化F_{ST}值计算之二

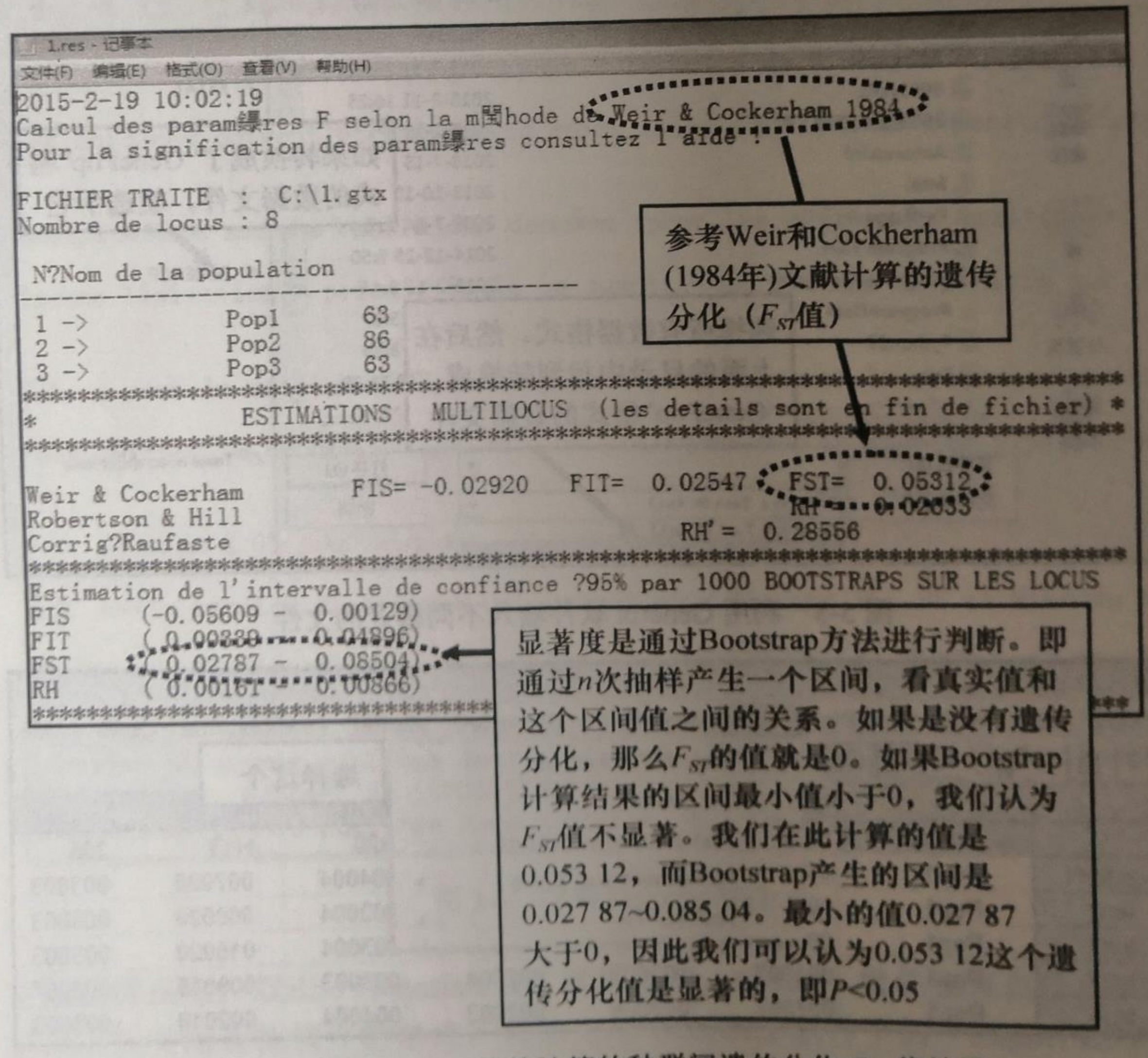

图5-6　利用Genetix软件计算的种群间遗传分化F_{ST}值结果

算完总的遗传分化值后，我们可以计算两两种群间的遗传分化（图 5-7，图 5-8）。由于刚才计算了总的遗传分化的值，文件被保存在了“1.res”文件中，现在又计算两两遗传分化的值，也要被保存在这个文件中，那么以前被保存的内容有可能被删除，因此软件提示“1.res”这个文件中以前的内容将被删除（图 5-9）。在此选“Oui”，表示删除以前的结果，因为前面计算的结果我们已经看过，不用了。读者也可以把以前的结果转移保存到其他文件夹下。

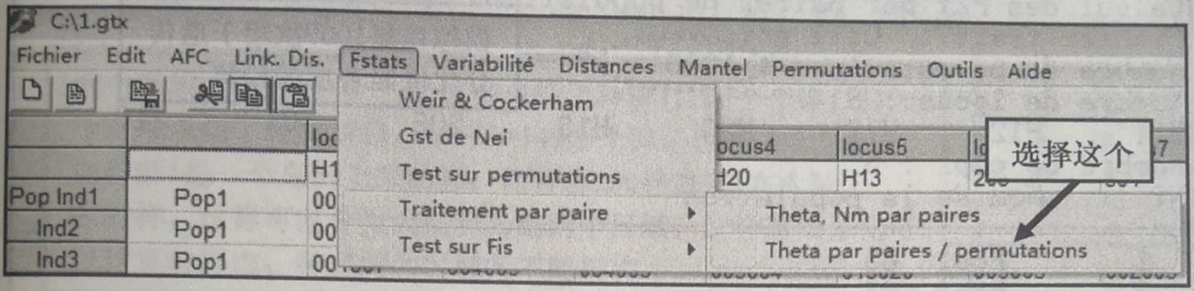

图 5-7 利用 Genetix 软件进行两两种群间遗传分化 F_{ST} 值计算之一

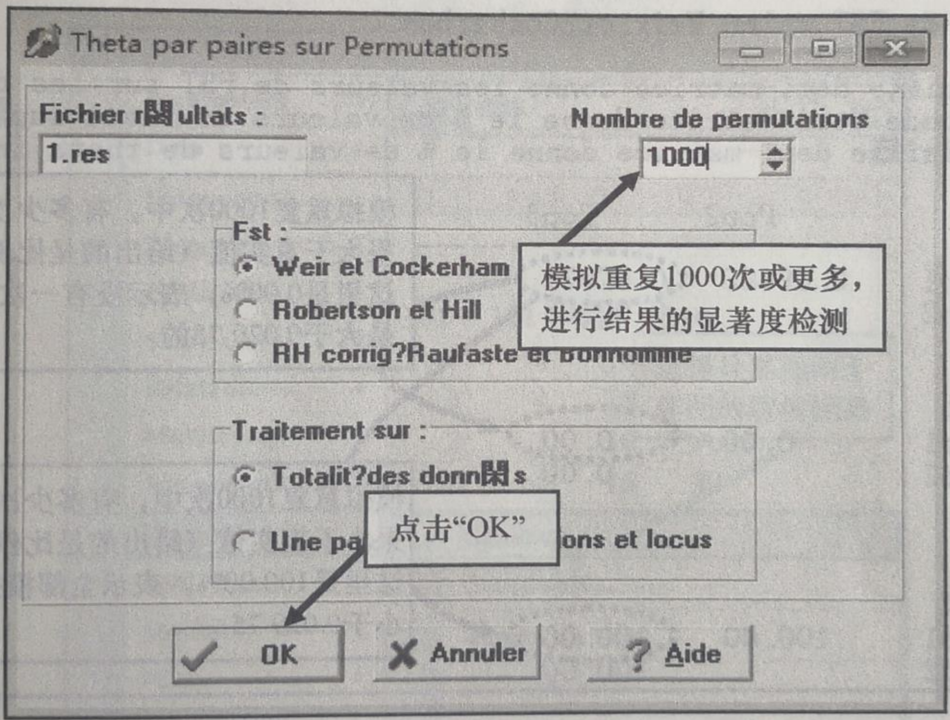

图 5-8 利用 Genetix 软件进行两两种群间遗传分化 F_{ST} 值计算之二

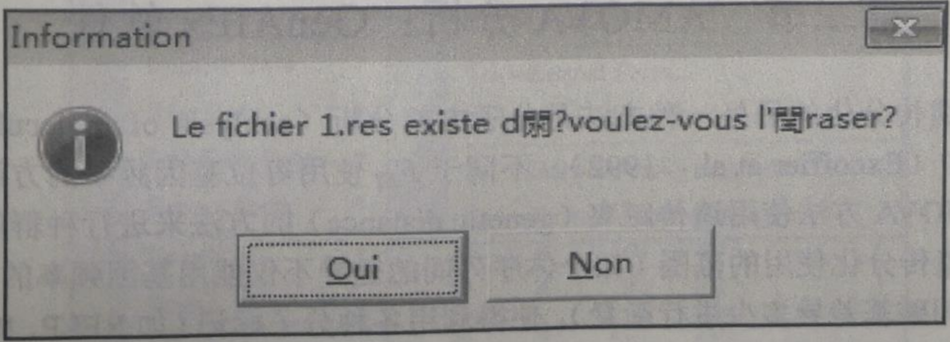

图 5-9 因为用同一文件名保存数据，Genetix 软件询问是否保留原有结果

照图 5-13～图 5-17 进行操作。值得说明的是，我们这里是用 GenAlEx 软件计算两两个体间的遗传距离，但也可以用其他方法算得的遗传距离进行 AMOVA 计算。另外就是 AMOVA 计算可以用 Arlequin 软件进行计算，但其操作较复杂些，不如 GenAlEx 软件操作方便。Arlequin 软件可从 http：//cmpg.unibe.ch/software/arlequin35/下载。

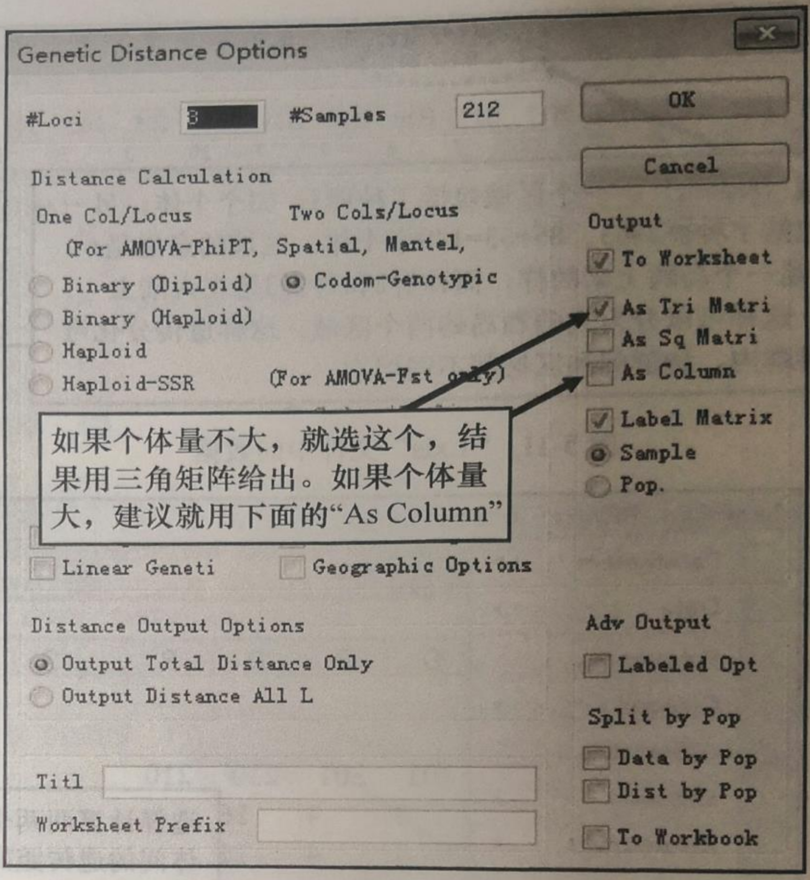

图 5-13 利用 GenAlEx 软件进行 Φ_{ST} 值计算之二

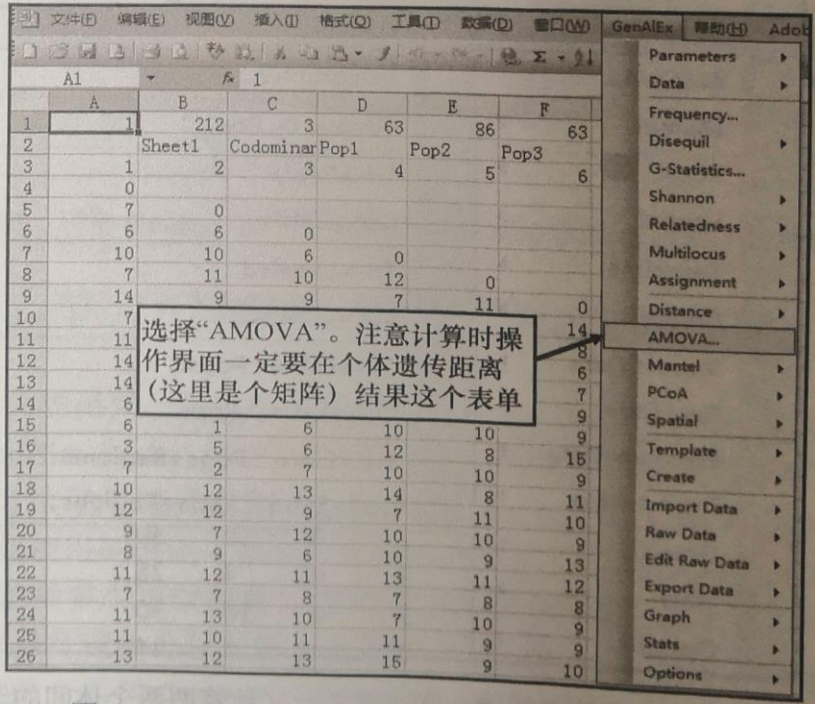

图 5-14 利用 GenAlEx 软件进行 Φ_{ST} 值计算之三

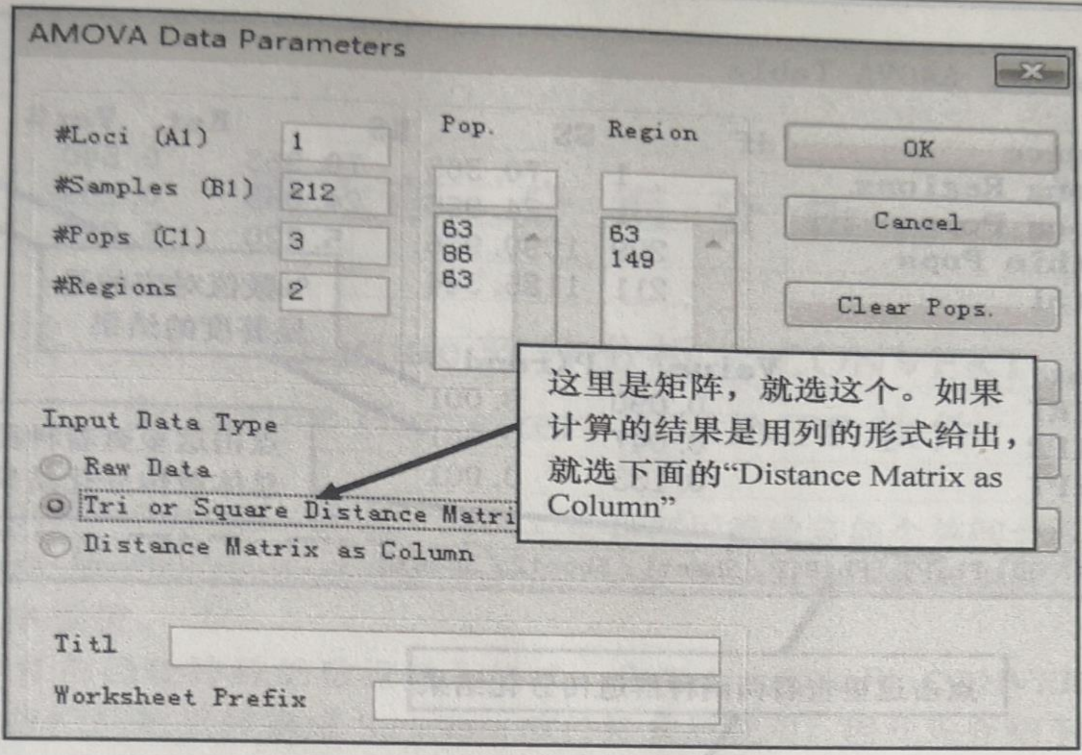

图 5-15　利用 GenAlEx 软件进行 Φ_{ST} 值计算之四

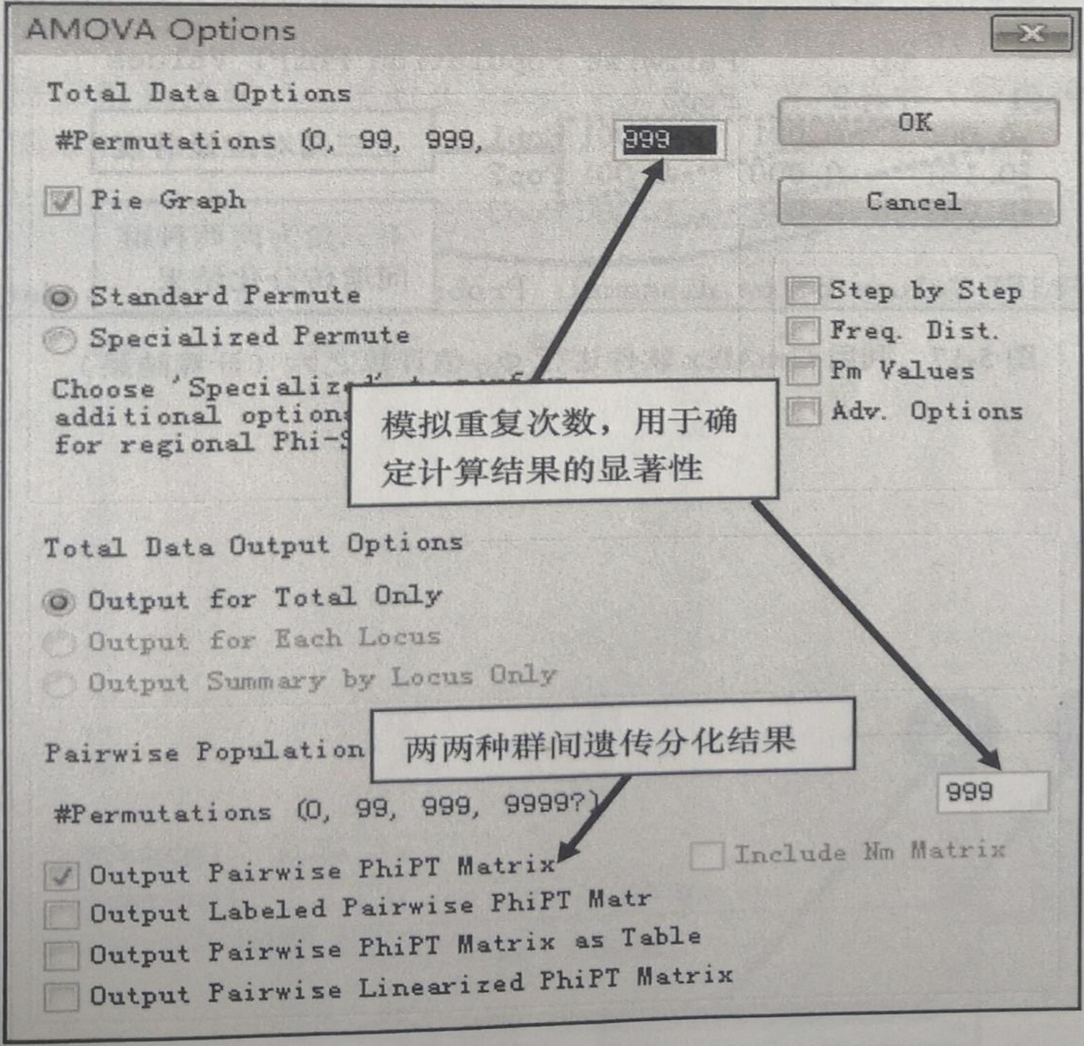

图 5-16　利用 GenAlEx 软件进行 Φ_{ST} 值计算之五

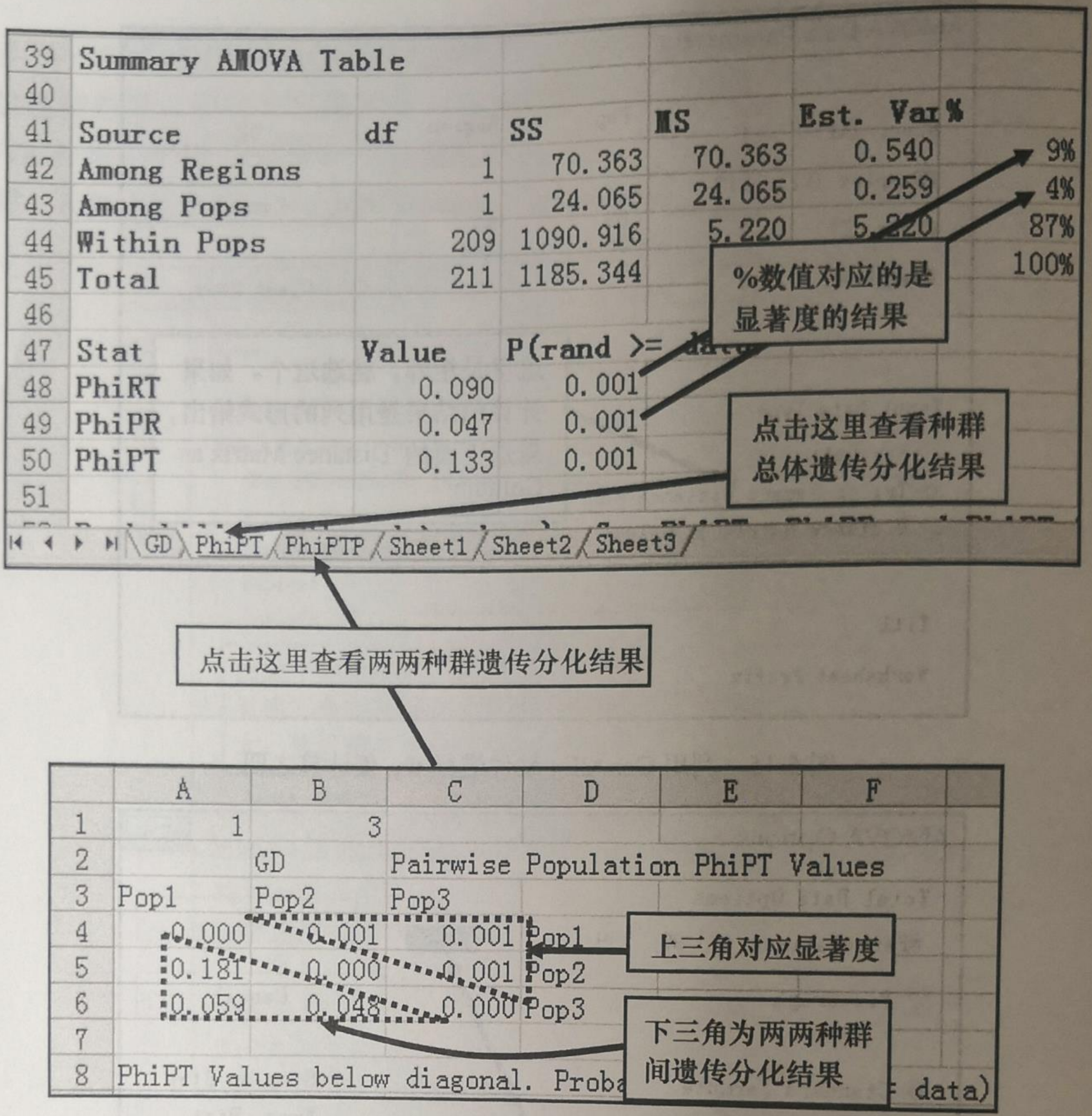

图 5-17　利用 GenAlEx 软件进行 Φ_{ST} 值计算之六（计算结果）
