# Supplementary material for: Genetic characteristics of P. falciparum parasites collected from 2012 to 2016 and anti-malaria resistance along the China-Myanmar border
Source: PLoS One. 2023 Nov 10;18(11):e0293590. doi: 10.1371/journal.pone.0293590 (PMC10637670; doi:10.1371/journal.pone.0293590)
Supplement: Attachment — Submitted filename: Response to Reviewers.docx [file pone.0293590.s003.docx]

**Response to Reviewers**

1. Line 206-225: There is no reason to have all the statistics (the 25% per = 2.74, per = 5.02, etc etc) reported in the main text—They are all already Table 2. I suggest using the main text to highlight only the key results.

***Response: Revised as reviewer’s suggestion***

2. Line 39: Missing a the: In (the) NW (north-west Yunnan…)

***Response: Revised as reviewer’s suggestion***

3. Line 47: Change phrasing: no difference in PCT50s were present between…

***Response: Revised as reviewer’s suggestion***

4. Line 47-50: having the actual values of He for each of the groups could be useful

***Response:*** According to reviewer’s suggestion, the median and mean He values of 4 groups instead of the description of high or low

5. Line 52: would change the word inferred to hypothesized—I am more used to being used in the statistical sense (ie for model selection or statistical parameter inference)

***Response: Revised as reviewer’s suggestion***

6. Line 63: backward economic development – would advise a rephrase to avoid offending people

***Response: According to reviewer’s suggestion, the word “backward” has been replaced with “low”***

7. Line 65: Malaria is one of infectious diseases – missing a the

***Response: Revised as reviewer’s suggestion***

8. Line 67: “last reported local case” instead of last report oflocal case

***Response: Revised as reviewer’s suggestion***

9. Line 68: Most of them (78.02, 4099/5254) were missing –missing a were

***Response: Revised as reviewer’s suggestion***

10. Line 70: The emergence and spread

***Response: Revised as reviewer’s suggestion***

11. Line 74: suggest replacing “because” with “following”

***Response: According to reviewer’s suggestion, “because of” in line 74 has been replaced with “following”***

12. Line 77-80: Rephrase as: In China, ACTs were used as the official first-line drugs to treat uncomplicated P. falciparum malaria starting in 2006.

***Response: Revised as reviewer’s suggestion***

13. Line 84: Rephrase: Treatment with DHA-PIP initially demonstrated prolonged parasite clearance time( PCT), but began to fail as the partner drugs began failing in the GMS around 2010.

***Response: Revised as reviewer’s suggestion***

14. Line 171: replace treated with examined

***Response: There was no word “Treated” in this line. I just deleted a extra space. I think reviewer mentioned should be Line 161. And the word “treated “ in this line has been replaced with “examined”***.

15. Line 306: Rephrase “ so surveillalnce sites are usually set in these Prefectures in those days.” The phrasing is too casual for a scientific paper in my opinion

***Response: According to reviewer’s suggestion, this sentence had revised to “So malaria patients were mainly enrolled in these Prefectures in those days.”***

16. Line 401: oppression is not the right word—advise selective pressure

***Response: Revised as reviewer’s suggestion***

17. Suggestions from editor 1

If applicable, we recommend that you deposit your laboratory protocols in protocols.io to enhance the reproducibility of your results. Protocols.io assigns your protocol its own identifier (DOI) so that it can be cited independently in the future. For instructions see: <https://journals.plos.org/plosone/s/submission-guidelines#loc-laboratory-protocols>. Additionally, PLOS ONE offers an option for publishing peer-reviewed Lab Protocol articles, which describe protocols hosted on protocols.io. Read more information on sharing protocols at <https://plos.org/protocols?utm_medium=editorial-email&utm_source=authorletters&utm_campaign=protocols>.

***Response: For protocols in this manuscript, I think 2 are most important. First was the guideline for calculating the values of He and Fst (in Chinese), and the second was test of Pfmdr1, PfPM2 and Pfβ-tubulin before calculating the copy number. They are both created reported by other researchers and almost no modifications have been made in our study. So I do not think they are worth of depositing in protocols. However, I attached the protocol here and gave some suggestions in the “Limitations” part. This might not only enhance the reproducibility of our results, but also could know the way to obtain more accurate results in readers’ study.***

1. ***The guideline for calculatiing He and Fst (in Chinese) will upload as attachment named “Guideline for calculating He and Fst (In Chinese)”***
2. ***The details of testing 3 genes are as followings:***

***Primers:*** Same with reference 38

***Reagent：***TB Green® Premix Ex Taq™ (Tli RNaseH Plus) purchased from Takara（RR420）

***qPCR machine:*** Bio-rad CFX96

***Reaction Condition***：95 ℃ for 3 min; 45 circles of 95 ℃ for 15 sec, 58 ℃ for 20 sec and Plate Read，72 ℃ for 20 sec; Melt Curve 65 ℃ to 95 ℃，increment 5 ℃ for 5 sec and Plate Read； End.

1. ***The sequencing and aligning of partial sequencs of PfM2C for isolates with low copy numbers of less than 1.***

```
Primers for Nest PCR of PfM2C gene are as followings
M2CFW	5'-CAATTCAACATTTGATGGATTAAAC-3’
M2CFN	5'-TTCCGAACATTTAACTATTGGATTT-3’
M2CR	5'-AACAATTGGATCTACTGAACCTAT-3’

Reagent：Premix Taq™ (TaKaRa Taq™ Version 2.0 plus dye) purchased from Takara（RR901）.
qPCR machine: Bio-rad C1000
```

***Reaction Condition***：95 ℃ for 3 min; 35 circles of 95 ℃ for 15 sec, 59 ℃ for 20 sec，72 ℃ for 20 sec; 72 ℃ for 5 min； 12℃ for ∞.

1. ***CLUSTAL multiple sequence alignment***

***Samples 20, 23, 26 from other study but also show low copy number as reviewer had mentioned. They are aligned with the sequence from isolate Pf 3D7 (XM_001348214). No difference presented among them including areas of primers (Red marked) designed to test and calculate copy number.***

```
20P             GACTTACTAAAACAAATTATTTAGGTAGTTCAAATGATAATATCGAATTAGTAGATTTCC
23P             GACTTACTAAAACAAATTATTTAGGTAGTTCAAATGATAATATCGAATTAGTAGATTTCC
26P             GACTTACTAAAACAAATTATTTAGGTAGTTCAAATGATAATATCGAATTAGTAGATTTCC
PFM2C           GACTTACTAAAACAAATTATTTAGGTAGTTCAAATGATAATATCGAATTAGTAGATTTCC
                ************************************************************

20P             AAAATATAATGTTTTATGGTGATGCAGAAGTTGGAGATAACCAACAACCATTTACATTTA
23P             AAAATATAATGTTTTATGGTGATGCAGAAGTTGGAGATAACCAACAACCATTTACATTTA
26P             AAAATATAATGTTTTATGGTGATGCAGAAGTTGGAGATAACCAACAACCATTTACATTTA
PFM2C           AAAATATAATGTTTTATGGTGATGCAGAAGTTGGAGATAACCAACAACCATTTACATTTA
                ************************************************************

20P             TTCTTGATACAGGATCTGCTAATTTATGGGTCCCAAGTGTTAAATGTACAACTGCAGGAT
23P             TTCTTGATACAGGATCTGCTAATTTATGGGTCCCAAGTGTTAAATGTACAACTGCAGGAT
26P             TTCTTGATACAGGATCTGCTAATTTATGGGTCCCAAGTGTTAAATGTACAACTGCAGGAT
PFM2C           TTCTTGATACAGGATCTGCTAATTTATGGGTCCCAAGTGTTAAATGTACAACTGCAGGAT
                ************************************************************

20P             GTTTAACTAAACATCTATATGATTCATCTAAATCAAGAACATATGAAAAAGATGGAACCA
23P             GTTTAACTAAACATCTATATGATTCATCTAAATCAAGAACATATGAAAAAGATGGAACCA
26P             GTTTAACTAAACATCTATATGATTCATCTAAATCAAGAACATATGAAAAAGATGGAACCA
PFM2C           GTTTAACTAAACATCTATATGATTCATCTAAATCAAGAACATATGAAAAAGATGGAACCA
                ************************************************************

20P             AAGTAGAAATGAATTATGTGTCAGGAACTGTTAGTGGATTTTTCAGTAAAGATTTAGTAA
23P             AAGTAGAAATGAATTATGTGTCAGGAACTGTTAGTGGATTTTTCAGTAAAGATTTAGTAA
26P             AAGTAGAAATGAATTATGTGTCAGGAACTGTTAGTGGATTTTTCAGTAAAGATTTAGTAA
PFM2C           AAGTAGAAATGAATTATGTGTCAGGAACTGTTAGTGGATTTTTCAGTAAAGATTTAGTAA
                ************************************************************

20P             CTGTTGGTAATTTATCTCTTCCATATAAATTTATTGAAGTAATAGATACTAATGGATTCG
23P             CTGTTGGTAATTTATCTCTTCCATATAAATTTATTGAAGTAATAGATACTAATGGATTCG
26P             CTGTTGGTAATTTATCTCTTCCATATAAATTTATTGAAGTAATAGATACTAATGGATTCG
PFM2C           CTGTTGGTAATTTATCTCTTCCATATAAATTTATTGAAGTAATAGATACTAATGGATTCG
                ************************************************************

20P             AACCAACTTATACTGCTTCAACATTTGATGGTATCCTTGGTT
23P             AACCAACTTATACTGCTTCAACATTTGATGGTATCCTTGGTT
26P             AACCAACTTATACTGCTTCAACATTTGATGGTATCCTTGGTT
PFM2C           AACCAACTTATACTGCTTCAACATTTGATGGTATCCTTGGTT
                ******************************************
```

1. ***minor comments***

Upon on the experiences of the test of Pfmdr1, PfPM2 and Pfβ-tubulin, we recommend pre-test for the PCR system, such as amplification efficiency (E) and new equation curve between copy number and 2^-∆t^ values in new lab before sample test. These two data are recommended to obtain using DNA of isolate Pf 3D7 and /or synthetic fragments (introduced in reference 38) instead of plasmid DNA. The reason are temporarily attributed to the fragment being too short and the copy numbers of plasmid DNA are not accurate. Negative control (Pf 3D7) should be included in each run that might clear the difference of Ct values between different genes.

18. Suggestions from editor 2

Please review your reference list to ensure that it is complete and correct. If you have cited papers that have been retracted, please include the rationale for doing so in the manuscript text, or remove these references and replace them with relevant current references. Any changes to the reference list should be mentioned in the rebuttal letter that accompanies your revised manuscript. If you need to cite a retracted article, indicate the article’s retracted status in the References list and also include a citation and full reference for the retraction notice.

***Response: Revised as reviewer’s suggestion. One missed reference was added [40], one was deleted[48], one was supplemented [69].***

19. Please upload a copy of Figure 1 which you refer to in your text on page 9. Or if the figure is no longer to be included as part of the submission please remove all reference to it within the text.

Please include a figure label and title for Figure 1 in your main manuscript

***Response: “Fig.1 Flow chart of sample test” has been uploaded and the name has been included in the main manuscript.***

20. It appears that your ORCiD iD has not been validated in your Editorial Manager account and we are unable to proceed until that step is complete.

***Response: Revised as reviewer’s suggestion***
